# Unilateral Posterior Stabilization in Adult Spinal Pathologies: Comparative Clinical, Radiological, and Complication Outcomes of Dynamic Versus Rigid Systems

**DOI:** 10.3390/medicina61111958

**Published:** 2025-10-31

**Authors:** Uzay Erdogan, Ege Anil Ucar, Feride Bulgur Balay, Gurkan Berikol, Ibrahim Taha Albas, Mehmet Yigit Akgun, Tunc Oktenoglu, Ali Fahir Ozer, Ozkan Ates

**Affiliations:** 1 Bakırköy Prof. Dr. Mazhar Osman Training and Research Hospital, Istanbul 43606, Turkey; drferide.bulgur@gmail.com; 2 Department of Neurosurgery, Koç University, Istanbul 34010, Turkey; eucar17@ku.edu.tr (E.A.U.); myigitakgun@gmail.com (M.Y.A.); tuncoktenoglu@gmail.com (T.O.); alifahirozer@gmail.com (A.F.O.); atesozkan@hotmail.com (O.A.); 3 Department of Neurosurgery, Health Sciences University, Sincan Training and Research Hospital, Ankara 06949, Turkey; dr.berikol@gmail.com; 4 Istanbul Physical Therapy and Rehabilitation Training and Research Hospital, Istanbul 43606, Turkey; tahaalbas@gmail.com; 5 Spine Center, Koc University Hospital, Istanbul 43606, Turkey; 6 Koc University Research Center for Translational Medicine, Biomechanics and Endurance Laboratory, Istanbul 43606, Turkey; 7 Bioengineering and Orthopaedic Surgery Colleges of Engineering and Medicine, University of Toledo, Toledo, OH 43606, USA

**Keywords:** posterior spinal instrumentation, unilateral fixation, pseudoarthrosis, dynamic stabilization, adjacent segment disease, treatment outcome

## Abstract

*Background and Objectives*: Unilateral spinal stabilization has emerged as a less invasive alternative to bilateral fixation in the management of lateralized spinal pathologies. While both rigid and dynamic systems are utilized, comparative data regarding their clinical efficacy, radiological outcomes, and complication profiles—particularly in multilevel applications—remain limited. *Materials and Methods*: A retrospective, two-center analysis was conducted on 113 patients who underwent unilateral posterior spinal stabilization between 2019 and 2023. Patients were divided into unilateral rigid stabilization (URS, *n* = 41) and unilateral dynamic stabilization (UDS, *n* = 72) groups. Pathologies of the patients include disc herniations, foraminal and spinal stenosis, tumoral lesions and spondylolisthesis. Clinical outcomes were assessed using the Visual Analogue Scale (VAS) over a 24-month follow-up. Radiological parameters included fusion status, superior adjacent disc height, and foraminal height index. Complication rates, including adjacent segment degeneration (ASD), pseudoarthrosis, and screw loosening, were analyzed according to type-of-stabilization and construct length (two, three, or four levels). *Results*: Both URS and UDS groups demonstrated significant VAS improvement at final follow-up, with no significant differences between groups (*p* < 0.001). Fusion rates were significantly higher in the URS group (85.37% vs. 27.78%, *p* < 0.001), while pseudoarthrosis (39.02% vs. 16.62%, *p* = 0.081) were more frequent in URS. No cases of rod fracture or infection were observed. Complication rates, particularly ASD, increased with longer constructs (6.56%, 21.21%, vs. 31.58% *p* = 0.01), independent of stabilization type. *Conclusions*: Unilateral stabilization—whether rigid or dynamic—offers effective symptom relief with reduced surgical morbidity. However, dynamic systems may provide biomechanical advantages by preserving motion and minimizing adjacent segment stress. While rigid constructs yield higher fusion rates, they are associated with increased complications. These findings support the use of dynamic stabilization, particularly in multilevel constructs, and highlight the need for patient-specific surgical strategies to optimize outcomes and mitigate long-term complications.

## 1. Introduction

Posterior spinal stabilization is the cornerstone surgical treatment for treating a wide spectrum of spinal disorders including trauma, degenerative disease, neoplastic involvement, infection and deformities [1,2,3,4,5]. Bilateral rigid fixation using pedicle screws and rods remains the standard approach to restore stability, promote fusion, and relieve neurological compression. However, surgeons frequently encounter unilateral spinal pathologies in clinical practice, and unilateral decompressive procedures, such as facetectomies, may themselves induce iatrogenic segmental instability [6,7,8,9]. These scenarios create a distinct clinical demand for unilateral stabilization strategies.

Unilateral instrumentation has gained acceptance as a less invasive alternative in appropriately selected patients [10,11]. Reported advantages include shorter operative times, reduced blood loss, limited muscle dissection, decreased implant cost and less disruption of intact posterior elements, all while achieving satisfactory outcomes in many cases [12,13]. Nevertheless, the adequacy of unilateral constructs, especially when applied to multiple levels, remains a subject of debate. Concentrated loading on a single-sided construct may increase the risk of hardware failure and screw loosening [14]. Furthermore, the long-term implications of unilateral stabilization with respect to adjacent segment degeneration, proximal junctional kyphosis, and pseudoarthrosis are less well established compared to bilateral approaches.

Dynamic stabilization has emerged as a technique designed to overcome some of the limitations of rigid fusion. By allowing controlled motion at the instrumented segments, dynamic systems distribute mechanical stress more physiologically and may reduce the incidence of adjacent segment degeneration and other fusion-related complications [15,16]. While these systems have demonstrated encouraging outcomes in motion preservation and clinical outcomes, questions remain about their durability, especially in unilateral stabilization and multilevel applications.

Given these considerations, a direct comparison of unilateral rigid stabilization (URS) and unilateral dynamic stabilization (UDS) can provide valuable insights. In this study, we aimed to evaluate the efficacy, complications, clinical and radiological outcomes of UDS compared to URS over a two-year follow-up period. Additionally, we analyzed outcomes according to construct length to better define the risks and benefits of multilevel unilateral fixation. Finally, we compared fusion rates between URS and UDS in order to assess not only the stability achieved by rigid constructs but also the motion-preserving potential of dynamic systems.

## 2. Materials and Methods

### 2.1. Patient Selection

Patients who underwent unilateral spinal stabilization between 2019 and 2023 at Koç University Hospital, Istanbul, and Bakirkoy Prof. Dr. Mazhar Osman Neurology, Neurosurgery and Psychiatric Hospital, Istanbul, were retrospectively examined. Inclusion criteria were a minimum of two years of clinical and radiological follow-up. Patients with incomplete follow-up data or less than two years of follow-up were excluded. A total of 113 patients who received either unilateral dynamic or unilateral rigid stabilization were analyzed. The choice of stabilization system (rigid vs. dynamic) was determined according to the operating surgeon’s preference. The levels of instrumentation were selected based on the location of pathology and the segments involved.

### 2.2. Clinical and Radiological Evaluation

Diagnosis was established through detailed patient history, neurological examination, and radiological assessment. All patients underwent standing anteroposterior and lateral dynamic radiographs, computed tomography (CT), and magnetic resonance imaging (MRI). Recorded variables included demographic characteristics, operated level(s), type of stabilization, system used, and primary diagnosis.

Clinical outcomes were evaluated using the Visual Analogue Scale (VAS), with follow-up data collected for at least 2 years. VAS is a validated, 10-point subjective measure of pain intensity, where 0 indicates no pain and 10 represents the worst imaginable pain [17]. VAS scores were recorded preoperatively and at 6-, 12-, and 24-month postoperative follow-ups to assess resting pain levels over time. Radiological parameters included foraminal height index, and superior adjacent disc height. The foraminal height index represents the ratio of the neural foramen height to the vertebral body height, reflecting potential nerve root compression [18]. The superior adjacent disc height measures the vertical distance between the adjacent endplates above the instrumented segment, indicating preservation of adjacent disc integrity [19]. Complications assessed were adjacent segment disease (ASD), instrument failure, pseudoarthrosis, and postoperative infection. ASD was defined as radiographic degenerative changes at levels adjacent to the instrumentation accompanied by corresponding clinical findings such as pain and/or neurological deficits [20]. Preoperative, early postoperative, and 24-month postoperative computed tomography (CT) scans were obtained for all patients and used for comprehensive radiological assessment. Fusion status and pseudoarthrosis was evaluated by CT at the 24th postoperative month. Spinal fusion was defined as the presence of continuous trabecular or cortical bone bridging across the instrumented intervertebral space on computed tomography (CT), with no visible radiolucent gap or motion between vertebral bodies. Pseudoarthrosis was defined as the absence of solid osseous continuity, the presence of a persistent radiolucent cleft, halo formation, failure of the instrumentation, e.g., screw loosening, or motion at the fusion site on CT images. Any case demonstrating screw loosening or halo formation around implants was classified as pseudoarthrosis, even when partial bone fusion was observed, since loosening indicates micro-instability and incomplete load sharing across the fusion mass [21,22]. All patients underwent preoperative and postoperative standing anteroposterior and lateral dynamic radiographs to evaluate implant placement and spinal alignment.

Spinal instability was defined through a combination of clinical and radiological criteria. Clinically, it was suspected in patients presenting with mechanical axial pain aggravated by activity and relieved with rest. Radiological indicators included: (1) sagittal translation >3 mm or segmental angulation >10° on flexion–extension radiographs; (2) CT findings such as vacuum phenomenon within the disc or facet joints, subchondral sclerosis, or hypertrophy of the ligamentum flavum; and (3) MRI evidence of facet joint effusion, hyperintensity, or Modic changes at adjacent endplates. A diagnosis of functional instability was established when these imaging findings were consistent with the clinical presentation [23,24,25,26,27].

### 2.3. Surgical Technique

The surgical decompression strategy was tailored to the underlying pathology, its location and the extent of stabilization required. For patients with disc herniations, tumoral lesions, or foraminal stenosis, a unilateral construct was utilized. In cases of spinal stenosis or spondylolisthesis, unilateral laminotomy for bilateral decompression (ULBD) was performed. Stabilization was indicated in cases with radiological evidence of instability—including listhesis, vacuum phenomenon, or significant facet degeneration—or when intraoperative decompression resulted in iatrogenic instability [6]. For unilateral rigid stabilization, standard pedicle screws were placed through the Wiltse paraspinal approach at the affected levels. Titanium rods were then secured to achieve rigid fixation and promote segmental fusion. The posterolateral surface was decorticated, and local autograft harvested from decompression bone was applied to facilitate fusion. For the unilateral dynamic stabilization group, Safinaz DSS (Dynamic screw, Medikon Tipsan AS, Bornova, Turkey) screws were inserted into the targeted pedicles in the same manner, after which polyetheretherketone (PEEK) or helical rods were applied to permit controlled motion while maintaining stability. In cervical cases, lateral mass screws were used for fixation at levels C3–C6. All procedures were performed under intraoperative fluoroscopic guidance to confirm accurate screw positioning and rod alignment.

### 2.4. Statistical Analyses

All statistical analyses were performed using Prism version 10.0 (GraphPad Software, LLC, Boston, MA, USA). Data normality was assessed using the Shapiro–Wilk test. Normally distributed variables are reported as the mean ± SD and categorical variables are reported as the number (percentage). Continuous variables were analyzed using Mann–Whitney U and Kruskall-Wallis for two-group and multi-group analyses, respectively. Categorical variables were analyzed by Chi-Square test. To indicate significance, *p* < 0.05 was considered.

## 3. Results

**Figure 1:**
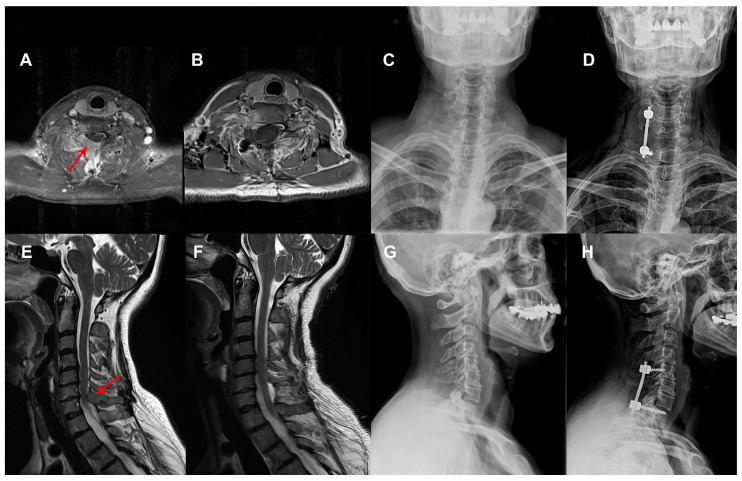
A 54-year-old female patient, recently diagnosed with breast cancer, presented with new-onset neck pain, right arm pain, and right arm weakness, most pronounced in elbow extension (motor strength: 2/5). Preoperative imaging includes (**A**) T1-weighted axial MRI and (**E**) T2-weighted sagittal MRI demonstrating a mass lesion at the C6–C7 level (Arrow) causing significant spinal cord compression. Preoperative cervical radiographs ((**C**): anteroposterior; (**G**): lateral views) show preserved vertebral alignment. The patient underwent mass excision and unilateral rigid stabilization from right C5 to T1. Postoperative images (**B**) T1-weighted axial MRI and (**F**) T2-weighted sagittal MRI reveal gross total resection of the mass with satisfactory spinal cord decompression. Postoperative radiographs ((**D**): anteroposterior; (**H**): lateral views) demonstrate appropriate placement of the unilateral rigid construct. Following surgery, the patient experienced complete resolution of neck and arm pain, and motor strength at elbow extension improved to 4/5. Histopathological analysis confirmed the lesion to be consistent with metastatic breast carcinoma.

**Figure 2:**
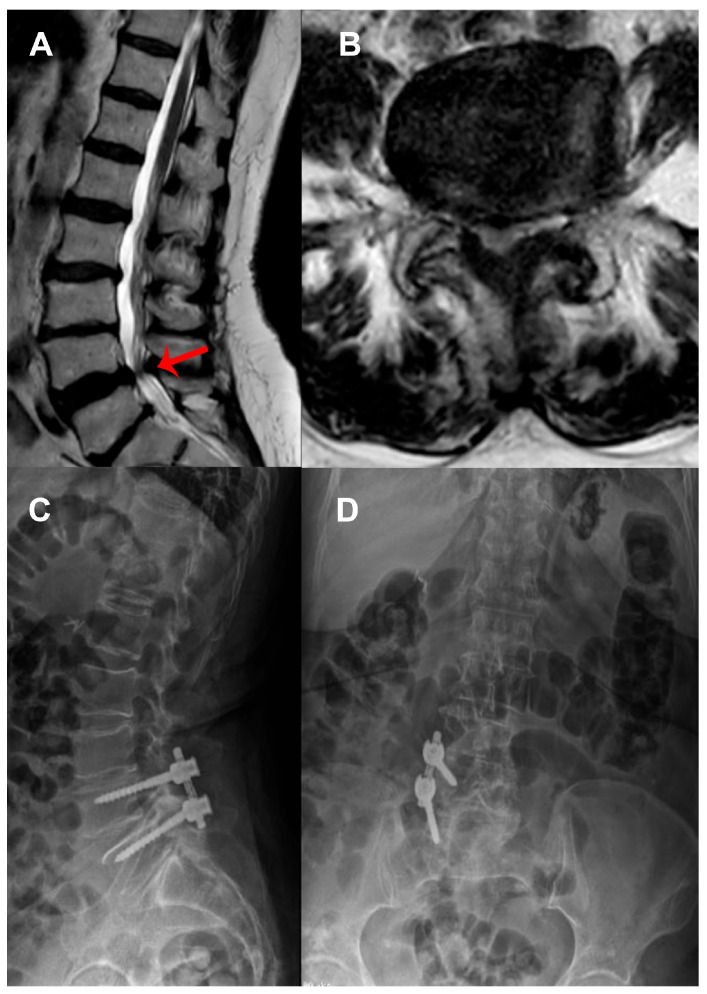
A 71-year-old female patient presented with worsening lower back and posterolateral leg pain, weakness in right ankle dorsiflexion, accompanied by neurogenic claudication after walking 50 m. (**A**) Sagittal and (**B**) axial T2-weighted MRI demonstrating spinal stenosis (arrow) at the L4–L5 level. The patient underwent unilateral laminotomy for bilateral decompression and right-sided L4–L5 stabilization using dynamic screws with a helical rod. Postoperative standing radiographs: (**C**) lateral and (**D**) anteroposterior views, confirming correct alignment with a dynamic construct.

**Figure 3:**
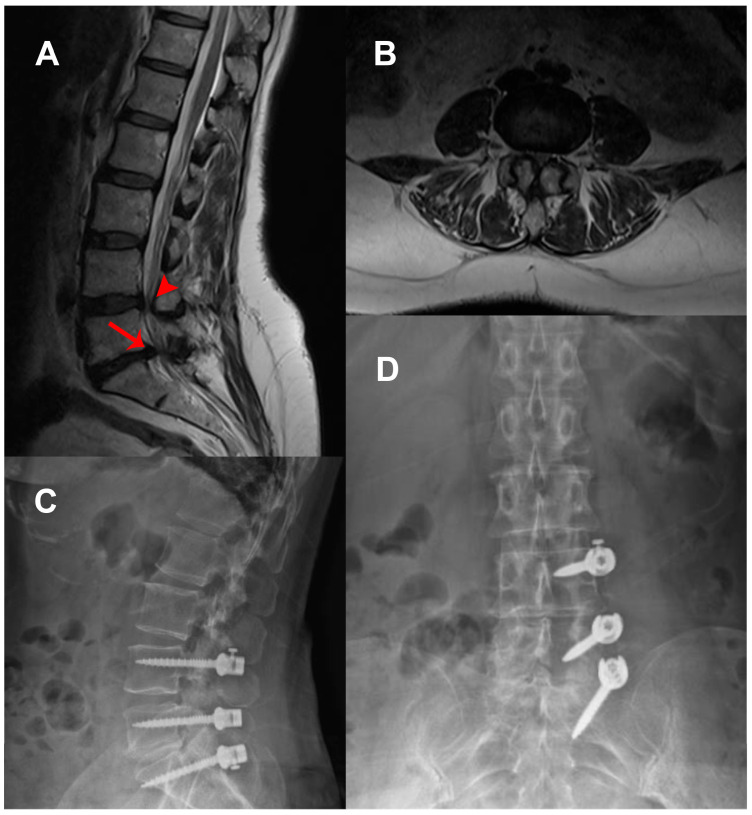
A 54-year-old female patient presented with new-onset lower back pain and bilateral leg pain, more pronounced on the left, accompanied by left leg hypoesthesia. (**A**) Sagittal and (**B**) axial T2-weighted MRI demonstrating spinal stenosis (arrowhead) at L4–L5 and disc herniation (arrow) at L5–S1. The patient underwent L4–S1 decompression and L5–S1 microdiscectomy, followed by left-sided L4–S1 stabilization using dynamic screws with a PEEK rod. Postoperative standing radiographs: (**C**) lateral and (**D**) anteroposterior views confirming stable dynamic fixation.

A total of 113 patients who underwent unilateral posterior spinal stabilization between 2019 and 2023 were retrospectively analyzed. The cohort consisted of 54 females (47.79%) and 59 males (52.21%), with a mean age of 59.65 ± 11.84 years. Sixty-one patients (53.98%) underwent two-level stabilization, 33 patients (29.20%) underwent three-level stabilization, and 19 patients (16.81%) underwent four-level stabilization. Dynamic stabilization (UDS) was performed in 72 patients (63.72%), while rigid stabilization (URS) was used in 41 patients (36.28%) (Table 1). The mean duration of clinical follow-up was 33.8 ± 12.44 months (range, 24–69 months). The distribution of underlying pathologies and operated levels are presented in Table 2. Selected cases from this cohort are presented in Figure 1, Figure 2 and Figure 3.

### 3.1. Clinical Outcomes

Visual Analogue Scale (VAS) scores improved significantly across the cohort, decreasing from a mean preoperative score of 8.33 ± 1.46 to 2.76 ± 1.81 at 24-month follow-up (*p* < 0.001). Improvements were observed across all subgroups, regardless of stabilization type or number of operated levels. The mean reduction in VAS (ΔVAS) did not differ significantly between UDS and URS groups (*p* = 0.59) or between patients with two-, three-, or four-level constructs (*p* = 0.97) (Table 3). Similarly, there were no significant difference between preoperative, 6th month, 12th month and 24th month postoperative VAS values both according to level and type of stabilization (level of stabilization: *p* = 0.07, *p* = 0.47, *p* = 0.19, *p* = 0.16; type of stabilization: *p* = 0.52, *p* = 0.61, *p* = 0.85, *p* = 0.08, respectively).

### 3.2. Radiological Outcomes

Superior adjacent disc height (SADH) and foraminal height index were evaluated to assess adjacent segment changes. In terms of type of stabilization, there were no significant difference between preoperative, early-postoperative and 24th month postoperative values, *p* = 0.08, *p* = 0.50, *p* = 0.85 respectively. Patients in the UDS group demonstrated a significant increase in SADH at early-postoperative imaging (*p* = 0.03), whereas the URSgroup showed significant deterioration in SADH by 24 months (*p* = 0.01) (Table 4). No significant differences were detected in SADH changes when stratified by level of stabilization, *p* = 0.89. Similarly, in terms of level of stabilization, no significant difference exist between preoperative, early-postoperative, nor 24th month postoperative SADH values, *p* = 0.52, *p* = 0.61, and *p* = 0.85 respectively. Foraminal height index remained stable in both groups, with no significant differences according to either stabilization type, *p* = 0.98, nor number of operated levels, *p* = 0.49.

### 3.3. Fusion and Pseudoarthrosis

Fusion rates were significantly higher in the URS group compared to the UDS group (85.37% vs. 27.78%, *p* < 0.001) (Table 5). Conversely, pseudoarthrosis was more common in the URS group (39.02% vs. 16.67%, *p* < 0.01). When stratified by construct length, neither fusion rates (*p* = 0.61) nor pseudoarthrosis rates (*p* = 0.97) differed significantly across two-, three-, and four-level groups (Table 6).

### 3.4. Complications

Seventeen patients (15.04%) developed adjacent segment disease (ASD) during follow-up. The incidence of ASD was significantly lower in patients who underwent two-level unilateral stabilization compared to those with three- or four-level constructs (*p* = 0.01). No significant difference was observed between three- and four-level stabilization groups (*p* = 0.51). Importantly, no postoperative infections or wound-related revisions were recorded, and no patient required revision surgery for mechanical failure or clinical deterioration. No rod fractures were observed in this cohort. Perioperative transfusion requirements did not differ significantly between two-, three- and four-level stabilization groups (*p* = 0.19).

## 4. Discussion

In this two-center cohort of 113 patients, unilateral stabilization, rigid or dynamic, produced comparable, clinically meaningful VAS improvement at 24 months; rigid constructs achieved higher CT-defined fusion, whereas dynamic constructs better preserved adjacent disc height, and ASD risk rose with longer constructs. These patterns align with meta-analyses showing that unilateral pedicle-screw constructs deliver similar fusion and clinical outcomes to bilateral fixation while reducing operative burden, with no consistent differences in VAS/ODI or overall peri/post-operative complications [28,29]. Our finding that dynamic systems match pain relief but do not clearly outperform rigid fusion echoes evidence that pedicle-based dynamic stabilization yields similar clinical outcomes and mixed effects on radiographic endpoints versus fusion, with uncertain superiority for preventing ASD [30,31]. Finally, the observed increase in ASD with greater construct length is consistent with the literature identifying adjacent-segment degeneration/disease as a long-term trade-off of fusion and highlighting construct-related risk factors [32,33,34].

Unilateral spinal stabilization represents a technique increasingly favored for its ability to address lateralized spinal pathologies with less tissue disruption, reduced operative time, and lower implant burden compared to bilateral constructs [35,36]. This approach aligns well with contemporary surgical trends emphasizing minimal invasiveness, particularly in degenerative and oncologic pathologies where unilateral disease predominates. In the present study, both unilateral dynamic stabilization (UDS) and unilateral rigid stabilization (URS) were associated with statistically significant improvements in pain scores over a 24-month period, consistent with prior literature demonstrating favorable clinical outcomes with unilateral instrumentation across various spinal levels and indications [10,11,37,38]. The lack of significant differences in VAS reduction between the UDS and URS groups underscores the comparable efficacy of both modalities, despite their distinct biomechanical principles. Furthermore, the finding that the number of operated levels did not negatively affect pain outcomes supports the versatility of unilateral constructs even in multilevel applications—challenging the traditional preference for bilateral fixation in longer constructs and reinforcing the role of unilateral approaches in reducing surgical morbidity without compromising clinical efficacy.

The biomechanical and biological distinctions between URS and UDS likely account for their differing complication profiles. As expected, rigid stabilization provided a mechanically favorable environment for osseous fusion, resulting in a significantly higher rate of radiographic fusion. This is in line with the mechanical premise that rigid constructs minimize micromotion, thereby promoting osseous bridging across instrumented segments. However, the increased rigidity may also contribute to stress concentration at adjacent levels, accelerating adjacent segment degeneration (ASD) and increasing the risk of pseudoarthrosis. Indeed, in our cohort, both ASD and pseudoarthrosis were more frequently observed in the URS group, findings consistent with previously reported biomechanical and clinical studies [15,16,32,39]. In contrast, dynamic stabilization systems aim to balance stability with controlled segmental motion, allowing for partial load sharing and maintenance of more physiological motion at both the instrumented and adjacent levels [40]. This mechanism may explain the significantly better preservation of superior adjacent disc height observed in the UDS cohort, which likely reflects reduced segmental stress transmission and preservation of intervertebral hydration. Although screw loosening was more frequently noted in the URS group, the difference did not reach statistical significance; nevertheless, this trend may further reflect the higher load transfer experienced in rigid constructs.

An important observation in our analysis is the impact of construct length on complication rates. While clinical outcomes remained stable across constructs of varying length, the incidence of ASD increased significantly in longer constructs, particularly those spanning three or four levels. This pattern was evident even in the UDS group, suggesting that despite the theoretical benefits of dynamic motion preservation, increased construct length remains a key biomechanical stressor to adjacent segments. These findings are supported by several prior studies demonstrating that longer constructs reduce the number of mobile spinal units, thereby concentrating mechanical stress at the terminal segments and accelerating degenerative changes [32,41,42,43]. The preservation of motion segments plays a critical role in the long-term biomechanical balance of the spine, and its compromise—even with dynamic systems—may predispose to ASD. Thus, construct planning must balance the goals of achieving adequate stabilization and decompression while minimizing unnecessary segmental immobilization. In this context, the findings support a more conservative and patient-specific approach to construct length, potentially favoring shorter unilateral constructs when clinically feasible to reduce long-term mechanical complications.

Unilateral dynamic stabilization offers a safe and effective alternative to rigid fixation, providing comparable clinical outcomes with fewer complications. By preserving physiological motion and reducing stress on adjacent segments, UDS lowers the risk of pseudoarthrosis and adjacent segment degeneration. However, increased construct length remains a significant risk factor for ASD, even with dynamic systems, highlighting the importance of limiting fusion levels when possible.

There are several limitations to the study. Firstly, this study is suspect to limitations and biases inherent to its retrospective design. The decision between unilateral rigid and dynamic stabilization was based on surgeon preference rather than randomization, introducing potential selection bias. Although follow-up duration exceeded two years, longer-term outcomes such as late-onset adjacent segment disease or delayed hardware failure could not be fully assessed. Moreover, heterogeneity of the cohort might reduce the generalizability of the findings. The sample size, while adequate for subgroup analysis, may still limit the power to detect subtle differences in rare complications. Radiographic evaluation of fusion relied on CT at 24 months, which may underestimate pseudoarthrosis compared to longer-term dynamic assessment. Additionally, functional outcome measures beyond VAS, such as disability indices or patient satisfaction, were not captured, limiting the scope of clinical interpretation. Future prospective studies with larger cohorts are needed for better capturing subtle differences between unilateral dynamic and rigid stabilization.

The principal strength of this study lies in its comparative two-center cohort design, encompassing a relatively large and heterogenous patient population that reflects real-world surgical practice. The use of uniform imaging criteria and long-term (≥24-month) CT-based follow-up enhances the reliability of fusion and complication assessment. Moreover, the direct comparison of unilateral dynamic versus rigid stabilization across multilevel constructs provides novel insights into biomechanics-informed patient selection—an area with limited existing data. Finally, all surgeries were performed under standardized operative protocols by experienced spine surgeons, ensuring methodological consistency and strengthening the validity of clinical and radiological outcomes.

Future research on unilateral spinal stabilization should prioritize prospective, multicenter trials that stratify by pathology, construct length and level of stabilization to define when unilateral rigid vs. dynamic constructs best balance pain relief, motion preservation, and adjacent segment risk. Standardized outcome sets, such as combining CT-based fusion metrics, quantitative segmental ROM, and patient-reported measures, are needed, alongside finite-element/AI-assisted preoperative planning to predict screw loosening and optimize implant stiffness. In addition, future biomechanics-first studies should establish decision thresholds for adding or limiting levels, and incorporate cost-effectiveness analyses. Finally, bioactive/bioprinted scaffolds [44] may augment future unilateral constructs by promoting biologic bridging without excessive stiffness, and patient-specific printed lattices could tune load-sharing in future hybrid systems, combining benefits of both rigid and dynamic stabilization techniques.

## 5. Conclusions

Both unilateral rigid and dynamic stabilization techniques provided significant and comparable clinical improvements over a two-year follow-up period, supporting the viability of unilateral constructs in selected spinal pathologies. While rigid stabilization achieved higher fusion rates, it was associated with increased rates of adjacent segment degeneration and pseudoarthrosis. In contrast, dynamic stabilization demonstrated better preservation of adjacent disc integrity and a favorable complication profile, while maintaining physiological mobility of stabilized segments. These findings underscore the importance of tailoring stabilization strategies to individual patient pathology and suggest that unilateral dynamic systems may offer biomechanical and clinical advantages.

## Figures and Tables

**Table 1:** Patient Demographics.

		Number	%
Age, Mean ± SD		59.65 ± 11.84	
Gender	Female	54	47.79
	Male	59	52.21
Level-of-Operation	Two-level	61	53.98
	Three-level	33	29.20
	Four-level	19	16.81
Type of Stabilization	Dynamic Stabilization	72	63.72
	Rigid Stabilization	41	36.28

**Table 2:** Distribution of primary pathology and operated spinal region.

Pathology	Number	%
Disc Herniation	44	38.94
Foraminal Stenosis	17	15.04
Spinal Stenosis	23	20.35
Tumoral Lesion	15	13.27
Spondylolisthesis	14	12.39
**Region of Operation**		
Cervical	5	4.39
Thoracal	8	7.02
Thoracolumbar	8	7.02
Lumbar	74	64.91
Lumbosacral	18	15.79

**Table 3:** Clinical Outcomes (Visual Analogue Scores).

	Preop (Mean + SD)	6th Month (Mean + SD)	12th Month (Mean + SD)	24 Month (Mean + SD)	*p* *
**Level-of-Operation**					
Two-level	7.89 ± 1.15	2.79 ± 1.42	2.12 ± 1.34	2.07 ± 1.27	<0.001
Three-level	8.76 ± 1.22	3.12 ± 2.25	2.98 ± 2.10	2.73 ± 1.84	<0.001
Four-level	9.02 ± 1.48	3.41 ± 2.21	3.23 ± 2.19	3.16 ± 2.08	<0.001
**Type-of-** **Stabilization**					
UDS	8.11 ± 1.26	2.86 ± 1.94	2.46 ± 1.77	2.06 ± 1.38	<0.001
URS	8.72 ± 1.20	3.22 ± 1.87	2.72 ± 1.62	3.12 ± 1.66	<0.001

UDS: Unilateral Dynamic Stabilization. URS: Unilateral Rigid Stabilization. * Kruskal–Wallis test was performed for statistical analyses.

**Table 4:** Superior Adjacent Disc Height.

	Preop (mm)	Early-Postop (mm)	24th Month (mm)	*p* *
**Level-of-Operation**				
Two-level	12.08 ± 3.73	13.81 ± 2.62	10.94 ± 3.11	0.0162
Three-level	10.80 ± 3.19	13.06 ± 2.52	10.58 ± 2.04	0.0482
Four-level	11.59 ± 4.11	11.42 ± 2.72	10.31 ± 3.21	0.8081
**Type-of-** **Stabilization**				
UDS	10.83 ± 3.53	12.98 ± 2.53	10.50 ± 2.56	0.0011
URS	13.03 ± 3.67	13.57 ± 2.59	11.14 ± 2.80	0.0062

UDS: Unilateral Dynamic Stabilization. URS: Unilateral Rigid Stabilization. * Kruskal–Wallis test was performed for statistical analyses.

**Table 5:** Fusion Rates and Complication Rates according to type of stabilization.

Type-of-Stabilization	URS (*n*, %)	UDS (*n*, %)	*p* *
Adjacent Segment Disease	9 (21.95%)	8 (11.11%)	0.1212
Fusion Rates	35 (85.37%)	20 (27.78%)	<0.001
Pseudoarthrosis	16 (39.02%)	12 (16.67%)	0.0081

UDS: Unilateral Dynamic Stabilization. URS: Unilateral Rigid Stabilization. * Chi-Square test was performed for statistical analyses.

**Table 6:** Fusion Rates and Complication Rates according to level of operation.

Level-of-Operation	Two-Level (*n*, %)	Three-Level (*n*, %)	Four-Level (*n*, %)	*p* *
Adjacent Segment Disease	4 (6.56%)	7 (21.21%)	6 (31.58%)	0.0096
Fusion Rates	29 (47.54%)	17 (51.52%)	9 (47.37%)	0.6095
Pseudoarthrosis	13 (21.31%)	10 (30.3%)	5 (26.32%)	0.9664

* Fischer’s exact test was performed for statistical analyses.

## Data Availability

The datasets used and/or analyzed during the current study are available from the corresponding author upon reasonable request.
